# Cardiac amyloidosis mimicking acute coronary syndrome: a case report and literature review

**DOI:** 10.1093/ehjcr/ytaa325

**Published:** 2020-10-29

**Authors:** Huan T Nguyen, Chuyen T H Nguyen

**Affiliations:** 1 Department of Geriatrics and Gerontology, University of Medicine and Pharmacy at Ho Chi Minh City, 217 Hong Bang Street, Ward 11, District 5, Ho Chi Minh City 70000, Vietnam; 2 Department of Cardiology, Thong Nhat Hospital, 1 Ly Thuong Kiet, Ward 7, Tan Binh District, Ho Chi Minh city 70000, Vietnam; 3 Department of Dermatology, University of Medicine and Pharmacy at Ho Chi Minh City, 217 Hong Bang Street, Ward 11, District 5, Ho Chi Minh City 70000, Vietnam

**Keywords:** Cardiac amyloidosis, Acute coronary syndrome, Chest pain, Case report

## Abstract

**Background:**

Cardiac amyloidosis, a progressive cardiac disease, results from the accumulation of undegraded proteinaceous substrates in the extracellular matrix of the heart. It may present as acute coronary syndrome (ACS); therefore, a clear distinction remains challenging in clinical practice. We describe a case of cardiac amyloidosis mimicking ACS.

**Case summary:**

A 72-year-old man experienced chest discomfort for 2 days. He gradually developed dyspnoea during the preceding month. Electrocardiogram (ECG) showed sinus rhythm with right bundle branch block and low voltage. Echocardiography revealed concentric left ventricular thickening, biatrial dilation, and preserved ejection fraction with predominantly left ventricular basal hypokinesis. Serial testing of the cardiac biomarkers showed persistently increased high-sensitive cardiac troponin T levels and normal serum creatine kinase myocardial band levels. He was diagnosed with ACS with haemodynamic stability. However, coronary angiography demonstrated non-obstructive coronary arteries. Furthermore, significant macroglossia and periorbital purpura were noticed. Laboratory investigations revealed elevated serum immunoglobulin free light chain (FLC) kappa and lambda levels with an increased FLC ratio. Histological analysis of the biopsied abdominal skin confirmed amyloidosis.

**Discussion:**

Cardiac amyloidosis often presents as restrictive cardiomyopathy. The usual symptoms include dyspnoea and peripheral oedema. Chest pain may manifest rarely, leading to misdiagnosis as coronary artery disease. Some findings suggestive of cardiac amyloidosis include clinical signs such as amyloid deposits, dyspnoea, low ECG voltage, and basal-predominant hypokinesis with relative apical sparing in echocardiography. Serum FLC test and abdominal skin biopsy can confirm the diagnosis of amyloidosis when a myocardial biopsy is not feasible.

**Learning points**

- Chest pain may manifest in cardiac amyloidosis, leading to misdiagnosis as acute coronary syndrome.
- Cardiac amyloidosis often presents as a restrictive cardiomyopathy with clinical signs resulting from amyloid deposits; dyspnoea, low electrocardiogram voltage, and basal-predominant hypokinesis in echocardiography.
- Serum free light chain tests and abdominal skin biopsy can be used to confirm the diagnosis of amyloidosis when myocardial biopsy is not feasible

## Introduction

Cardiac involvement in systemic amyloidosis reflects an infiltrative heart disease known as cardiac amyloidosis. It can have heterogeneous clinical manifestations characterized by restrictive cardiomyopathy, heart failure with preserved ejection fraction, arrhythmia, and conduction block.^1^ Chest pain may occur on rare occasions, leading to misdiagnosis as coronary artery disease.^2^^,^^3^ We describe a case of cardiac amyloidosis mimicking acute coronary syndrome (ACS). We discuss the literature and suggest a clinical approach to differentiate the two disorders.

## Timeline

**Table:** 

Initial presentation	Effort dyspnoea, slight limitation but feeling well at rest No fever, no peripheral oedema
1 month later	Exercise-induced chest pain near the sternum for 2 days Worsening dyspnoea, marked limitation of physical activity Hence, admitted for evaluation
In-hospital: Day 1 At Emergency Department	Electrocardiogram: right bundle branch block and low voltage Echocardiography: concentric left ventricular thickening, biatrial dilation, and preserved ejection fraction with left ventricular basal > apical-predominant hypokinesis High-sensitive cardiac troponin (hs-cTnT): 388 pg/mL on admission and 467 pg/mL after 3 h (normal ≤ 24.9 pg/mL). Creatine kinase myocardial band (CK-MB): 9 U/L on admission and 13 U/L after 3 h (normal ≤ 24 U/L) Coronary angiography: non-obstructive coronary arteries
In-hospital: Day 2 At Department of Cardiology	Macroglossia, and periorbital purpura were noted Patient administered losartan 12.5 mg o.d., bisoprolol 1.25 mg o.d., furosemide 20 mg o.d., and isosorbide mononitrate 60 mg o.d.
In-hospital: Day 5	Free light chain (FLC) kappa: 1220 mg/L (normal 6.7–22.4 mg/L). FLC lambda: 87.7 mg/L (normal 8.3–27.0 mg/dL). Kappa-lambda ratio: 13.9 (normal 0.31–1.56). hs-cTnT: 295 pg/mL. CK-MB: 11 U/L Amyloid deposits in biopsied abdominal skin Chest pain was relieved by nitrate Continue medical treatment without dose titration
In-hospital: Day 7	Clinical status and physical activity were gradually improved
In-hospital: Day 9	The patient was discharged on stable condition
Post-discharge: Day 5	The patient suddenly expired at home

## Case presentation

A 72-year-old man, experiencing chest pain near the sternum for 2 days, was admitted to our hospital. He had no history of coronary artery disease, hypertension, dyslipidaemia, and other comorbidities. He gradually developed dyspnoea during the preceding month, with no fever or peripheral oedema.

**Figure 1:**
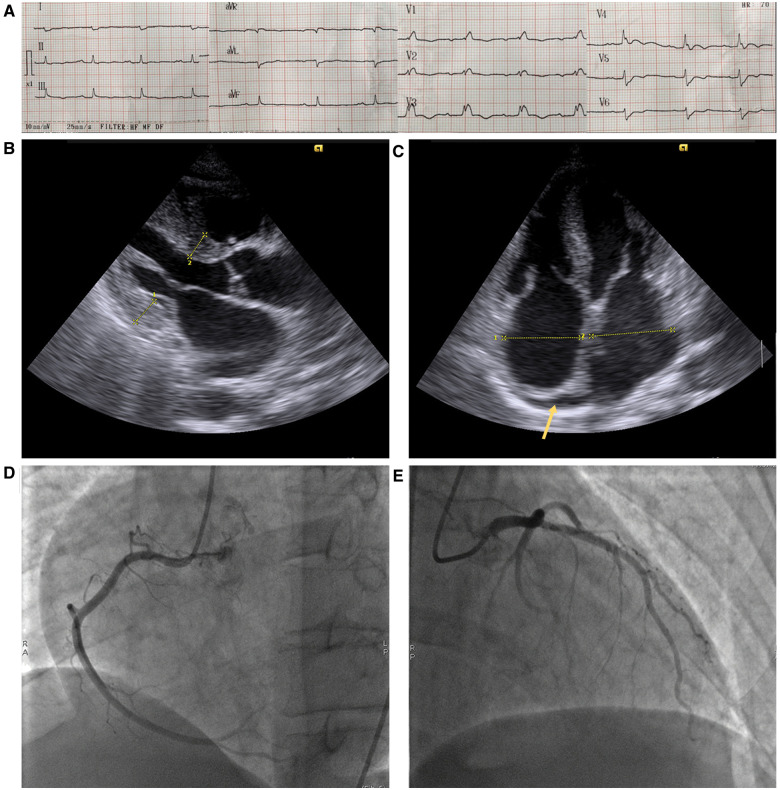
Electrocardiogram showing sinus rhythm with right bundle branch block and low voltage on the limb leads (*A*). On the parasternal long-axis view, echocardiography revealed concentric left ventricular hypertrophy with a thickened interventricular septum and posterior left ventricular wall (14 and 15 mm at the diastolic phase, respectively) (*B*). Four-chamber view echocardiography detected biatrial dilation and a mild pericardial effusion close to the right atrium (arrow) (*C*). Coronary angiography demonstrated non-obstructive right (*D*) and left (*E*) coronary arteries.

**Figure 2:**
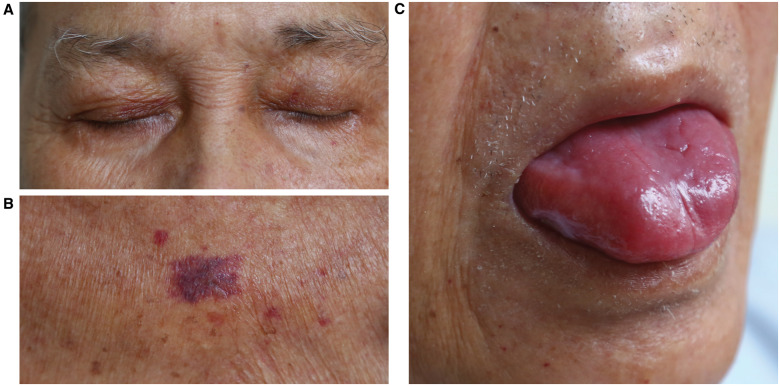
The patient had classic cutaneous involvement showing prominent purpuric lesions due to the deposition of amyloid in and around vascular structures, including periorbital ‘pinch purpura’ (*A*) and trunk (*B*). Macroglossia was also suggestive of a diagnosis of systemic amyloidosis (*C*).

**Figure 3:**
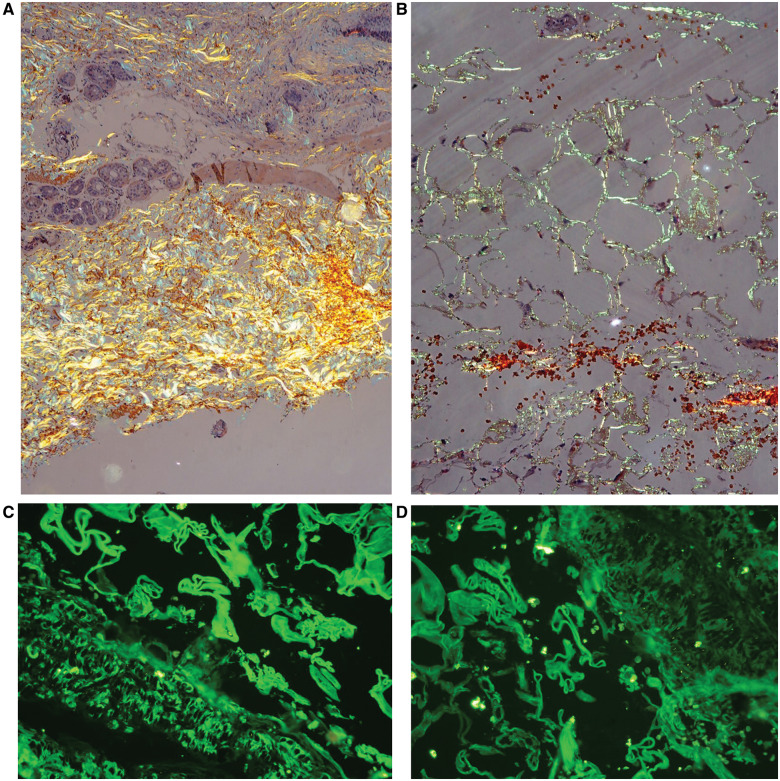
Congo red staining detected amyloid deposits on polarization microscopy, showing apple-green birefringence scattering through the subcutaneous layer (*A*, 200× magnification) and around the fat tissues (*B*, 400× magnification). Immunostaining showed a substantial accumulation of kappa (*C*) and lambda (*D*) light chain-immunoreactive amyloid in the subcutaneous layer.

On initial examination at the Emergency Department, the patient was haemodynamically stable (blood pressure of 110/65 mmHg; regular pulse rate of 70 b.p.m.; and respiratory rate of 20 breaths/min). Cardiac and respiratory examination showed normal heart sounds and an absence of murmurs, gallops, and clear lung fields. Electrocardiogram (ECG) showed sinus rhythm and low amplitude QRS complexes with right bundle branch block (RBBB) morphology (*Figure 1A*). Echocardiography revealed concentric left ventricular hypertrophy, biatrial dilation, mild pericardial effusion close to the right atrium (*Figure 1B and C*), and a preserved ejection fraction with predominantly left ventricular basal hypokinesis. Serial testing of cardiac biomarkers showed an increased level of high-sensitive cardiac troponin T (hs-cTnT), along with normal levels of serum creatine kinase myocardial band (CK-MB). According to these clinical and troponin findings, ACS had to be excluded, despite a normal level of CK-MB. The patient was given 500 mg of aspirin and 600 mg of clopidogrel before urgent percutaneous coronary intervention. However, coronary angiography demonstrated non-obstructive right and left coronary arteries (*Figure 1D and E*). The patient was referred to the Department of Cardiology for further evaluation, where screening for cardiomyopathy was performed. Physical re-examination showed signs of non-cardiac disease, suggesting amyloid fibril damage, including macroglossia, periorbital purpura, and purpura above the nipples (*Figure 2*). Laboratory investigations revealed elevated serum immunoglobulin free light chain (FLC) kappa and lambda levels with an increased FLC ratio. These findings were suggestive of amyloidosis; therefore, the histopathological features of an abdominal skin punch biopsy specimen were studied. Congo red stain detected amyloid deposits under the light microscope and showed apple-green birefringence using polarizing microscopy. Immunostaining showed accumulation of kappa light chain-immunoreactive amyloid (*Figure 3*). These histological analyses confirmed the diagnosis of immunoglobulin light chain (AL) amyloidosis in this patient.

During the 1 week of stay at our hospital, the patient’s medical management included losartan 12.5 mg o.d., bisoprolol 1.25 mg o.d., furosemide 20 mg o.d., and isosorbide mononitrate 60 mg o.d. Losartan and bisoprolol were indicated based on limited evidence in the treatment of heart failure with preserved ejection fraction.^4^ Furosemide and isosorbide mononitrate were used for maintaining euvolaemia and relieving the symptoms of chest discomfort, respectively.^1^ Because this regimen gradually ameliorated his clinical status, we decided to continue with the medical treatment. The patient was discharged in a stable condition. To screen for a possible concurrent multiple myeloma, the patient was referred to a haematology centre. Unfortunately, 5 days post-discharge, he suddenly expired at home.

## Discussion

Cardiac amyloidosis may present with symptoms of chest pain that need to be distinguished from a broad differential diagnosis. To improve the recognition of this disease in clinical practice, we present three points for discussion.

### Clinical characteristics of patients with cardiac amyloidosis presenting with chest pain

The prevalence of cardiac amyloidosis in patients with chest pain remains unclear. Approximately 15% of patients with AL amyloidosis experienced anginal pain with exertion.^2^^,^^3^ To further elucidate the characteristics of patients with cardiac amyloidosis presenting with chest pain, we performed a literature search of the PubMed database for articles published up to October 2019 with keywords including cardiac amyloidosis, chest pain, chest angina, angina pectoris, microvascular angina, and chest discomfort. We used the following search terms: ‘cardiac amyloidosis AND (chest pain OR chest angina OR angina pectoris OR microvascular angina OR chest discomfort)’. In addition to the PubMed database, we searched for potential reports from the reference lists of each included study manually; and scanned references of the relevant papers in PubMed and Google Scholar. Including our case, the clinical information of 16 sporadic patients with cardiac amyloidosis presenting with chest pain is shown in *Table 1*.

**Table 1:** Sporadic case report of cardiac amyloidosis presenting chest pain

Study	Saltissi *et al.*	Narang *et al.*	Ishikawa *et al.*	Ogawa *et al.*	Yamano *et al.*	Cantwell *et al.*	Whitaker *et al.*	Soma *et al.*	Sohn *et al.*	Tsai *et al.*	George *et al.*	Edwards *et al.*		Keller *et al.*	Adhikari *et al.*	Current patient
Year	1984	1993	1996	2001	2002	2002	2004	2010	2011	2011	2015	2015	2015	2016	2018	2020
Age	32	43	65	69	76	43	65	49	77	61	75	63	46	65	64	72
Sex	Male	Female	Male	Female	Female	Male	Male	Male	Male	Male	Male	Male	Male	Female	Male	Male
Type	AL	AL	AL	AL	AL	AL	AL	AL	ATTRwt	AL	AL	AL	ATTRm	AL	AL	AL
Chest pain	+	+	+	+	+	+	+	+	+	+	+	+	+	+	+	+
Dyspnoea	+	+	+	+	+	+	+	+	+	+	+	+	+	+	+	+
Troponin	NA	NA	Normal	NA	NA	↑	↑	↑	↑	NA	↑	↑	Normal	NA	↑	↑
CK-MB	NA	NA	Normal	NA	NA	Normal	NA	NA	NA	NA	NA	NA	NA	NA	NA	Normal
Serum FLC	NA	↑	NA	NA	NA	NA	NA	NA	NA	NA	↑	↑	NA	NA	↑	↑
ECG	LoV	LoV	AF	AVB	LoV	LoV	ST↓	LoV	LoV	NoV	ST↓	LoV	RBBB	NoV	LoV	LoV, RBBB
CAG	NA	Normal	Normal	Normal	Normal	Normal	Normal	Normal	Normal	Normal	Normal	Normal	NA	Normal	Normal	Normal
Echocardiography																
Restrictive pattern	NA	NA	NA	−	−	+	NA	−	+	+	+	+	+	+	+	+
Increased WT	+	+	NA	−	−	+	NA	−	+	−	−	+	+	+	+	+
Hypokinesis	+	+	NA	−	−	+	+	+	+	+	−	−	−	+	+	+
Histological evidence of amyloid deposition																
Myocardium	+	+	+	+	+	+	−	+	+	+	NA	+	+	+	NA	NA
Epicardial CA	NA	NA	−	−	−	−	−	−	−	−	NA	−	NA	NA	NA	NA
Small CA	+	+	+	+	+	+	+	+	+	+	NA	+	+	+	NA	NA
Non-cardiac	+	+	+	+	+	+	−	NA	NA	+	+	+	+	+	+	+

The reference list of sporadic cases is shown in Supplementary material online, *Table S2*.

AF, atrial fibrillation; AL, immunoglobulin light chain; ATTRm, mutant transthyretin; ATTRwt, wild-type transthyretin; AVB, atrioventricular block; CA, coronary arteries; CAG, coronary angiography; CK-MB, creatine kinase myocardial band; ECG, electrocardiogram; FLC, free light chain; LoV, low voltage in the limb leads; NA, not applicable; NoV, normal voltage in the limb leads; RBBB, right bundle branch block; ST↓, ST-segment depression; WT, wall thickness; +, present; −, absent; ↑, increased.

These data indicate that cardiac amyloidosis often presents as a restrictive cardiomyopathy, with clinical signs such as dyspnoea, low ECG voltage, and predominant basal hypokinesis in echocardiography. Serum FLC tests and abdominal skin biopsy can be used to confirm the diagnosis of amyloidosis when a myocardial biopsy is not feasible.

### Mechanism of chest pain in cardiac amyloidosis

Many diseases can cause chest pain. In patients with cardiac amyloidosis, this symptom might be due to exertion, mimicking coronary artery disease. However, previous studies have reported that patients with cardiac amyloidosis experience anginal pain but have no significant atherosclerotic epicardial coronary stenosis.^2^^,^^3^

According to Dorbala *et al*.,^5^ chest pain in amyloidosis involves three major mechanisms. First, structural: histopathology of the myocardium in patients with cardiac amyloidosis and anginal pain has demonstrated the accumulation of amyloid within the walls of the small coronary arteries, whereas the epicardial coronary arteries were normal with no amyloid deposition.^2^^,^^3^ Second, extravascular: perivascular and interstitial amyloid deposits might lead to extramural compression and reduced diastolic perfusion time. Additionally, myocardial necrosis was found in the myocardial areas surrounding the small vessels obstructed with amyloid infiltration, suggesting that obstruction of these small coronary arteries might lead to myocardial ischaemia.^6^ Third, functional: rest and vasodilator stress N^13^ ammonia positron emission tomography demonstrated coronary microvascular dysfunction. It showed significantly lower myocardial blood flow, lower coronary flow reserve, and higher minimal coronary vascular resistance in patients with cardiac amyloidosis than in normal volunteers and subjects with hypertensive left ventricular hypertrophy.^5^

The pathological factors causing accumulation of amyloid predominantly in small coronary vessels remain largely elusive. Other unknown factors still contribute to the mechanisms of chest pain in patients with cardiac amyloidosis.

### Awareness of cardiac amyloidosis when treating patients with chest pain

In clinical practice, while attending to a patient with chest pain, cardiologists have to first evaluate the potential life-threatening causes of chest pain, such as ACS, aortic dissection, pneumothorax, and pulmonary embolism. Once the acute aetiologies are excluded, it is reasonable to consider the possibility of amyloidosis in cases of ischaemic heart disease with a normal coronary angiogram. Our patient had elevated and stable hs-cTnT with ∼20% variation in troponin values and an unaltered ECG 3 h after admission, suggesting chronic myocardial injury.^7^ However, urgent coronary angiography was performed because this procedure should be considered when persistent ischaemic symptoms are noted in the presence of RBBB.^8^

Some imaging techniques have recently been shown to aid in the diagnosis of cardiac amyloidosis.^9^ Patients with cardiac amyloidosis present abnormal patterns of late gadolinium enhancement in both global transmural and subendocardial distributions on cardiac magnetic resonance imaging.^10^ In speckle-tracking echocardiography, impairments in strain that affect the basal areas more than the apex are a recognizable pattern for differentiating cardiac amyloidosis from other causes of left ventricular hypertrophy.^11^Technetium-labelled aprotinin or technetium-99m pyrophosphate scintigraphy can be used to identify cardiac amyloid.^12^

Systemic amyloidosis has a wide spectrum of non-specific clinical manifestations. Mucocutaneous findings are reportedly observed in ∼30–40% of patients and can be an early indicator of the disease. Our patient presented with classic cutaneous involvement for the diagnosis of systemic amyloidosis, including purpuric lesions, often present around the eyes, and macroglossia. This highlights the need to recognize cutaneous signs in the case of a systemic disease.^13^

## Conclusion

In the clinical approach to patients with chest pain, cardiac amyloidosis should be suspected once other common aetiologies, including ACS, are excluded. Diagnostic clues suggesting cardiac amyloidosis include unexplained dyspnoea, left ventricular hypertrophy with low ECG voltages, basal-predominant hypokinesis with apical sparing in echocardiography, and clinical features of amyloid deposition, particularly cutaneous findings. Evidence of amyloid in tissues is required to confirm the diagnosis of cardiac amyloidosis.

## Lead author biography

**Figure:**
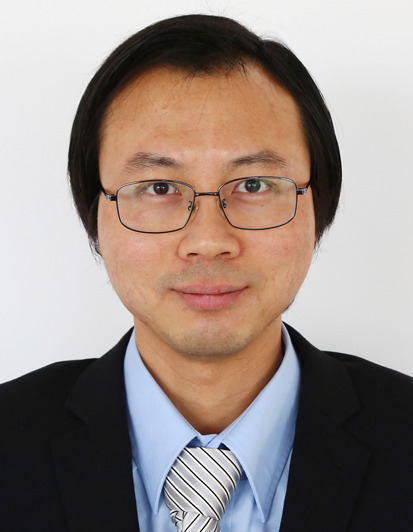


Dr Huan T. Nguyen is a clinical cardiologist as well as a lecturer in University of Medicine and Pharmacy at Ho Chi Minh City, Vietnam. He completed his PhD Program at Kansai Medical University, Osaka, Japan. His current research projects into the origin of cardiomyopathies and heart failure, which have contributed to improved management of patients with heart failure.

## Supplementary material

Supplementary material is available at *European Heart Journal - Case Reports* online.

## Supplementary Material

ytaa325_Supplementary_Data
