# Supplementary material for: Cardiac amyloidosis mimicking acute coronary syndrome: a case report and literature review
Source: Eur Heart J Case Rep. 2020 Oct 29;4(6):1–7. doi: 10.1093/ehjcr/ytaa325 (PMC7793193; doi:10.1093/ehjcr/ytaa325)
Supplement: ytaa325_Supplementary_Data [file ytaa325_supplementary_data.zip › ytaa325-suppl_data/15_EHJ-CR-D-20-00151R3_Slideset.pptx]

## Slide 1
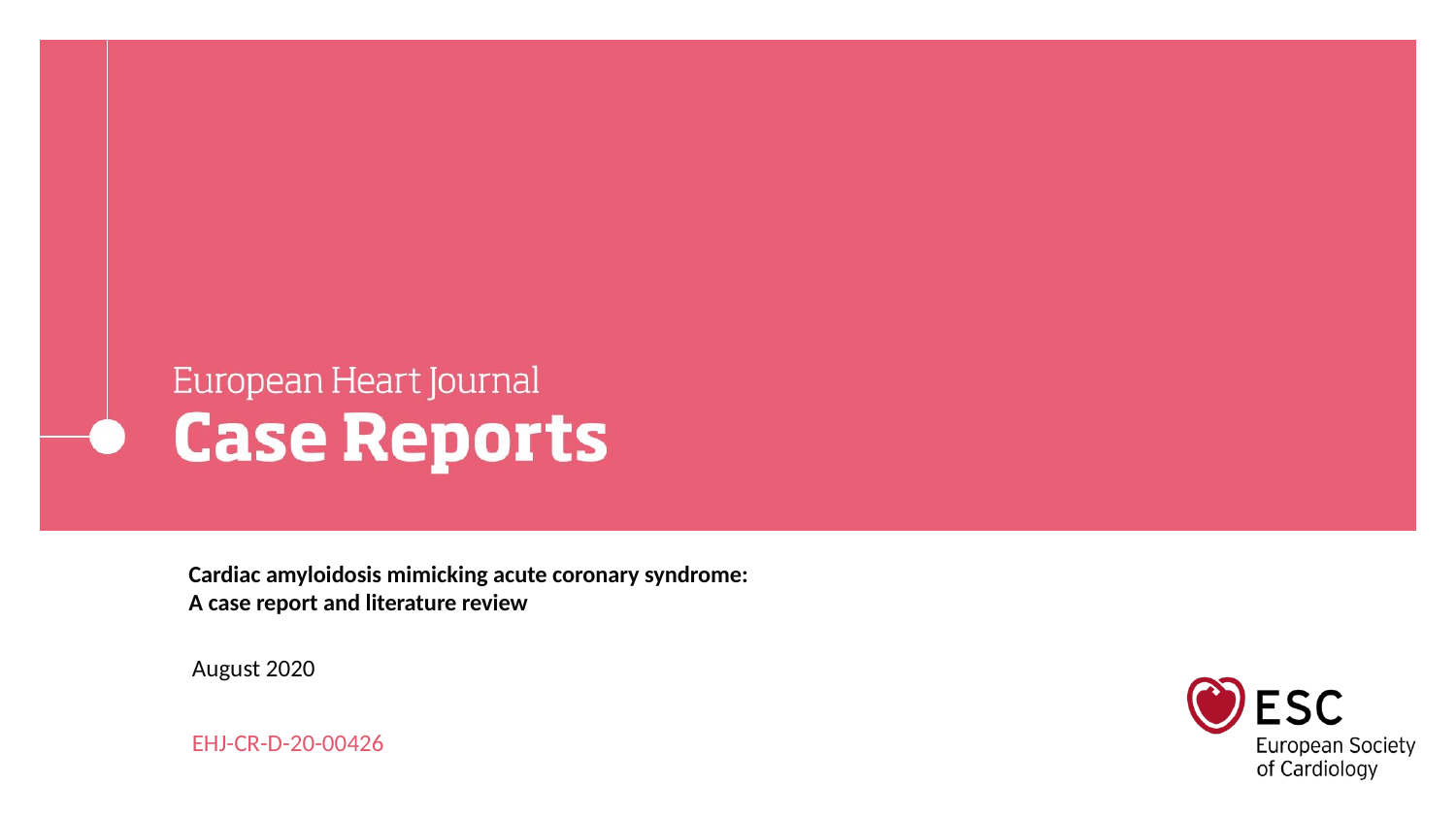

# Cardiac amyloidosis mimicking acute coronary syndrome: A case report and literature review
August 2020
EHJ-CR-D-20-00426

## Slide 2
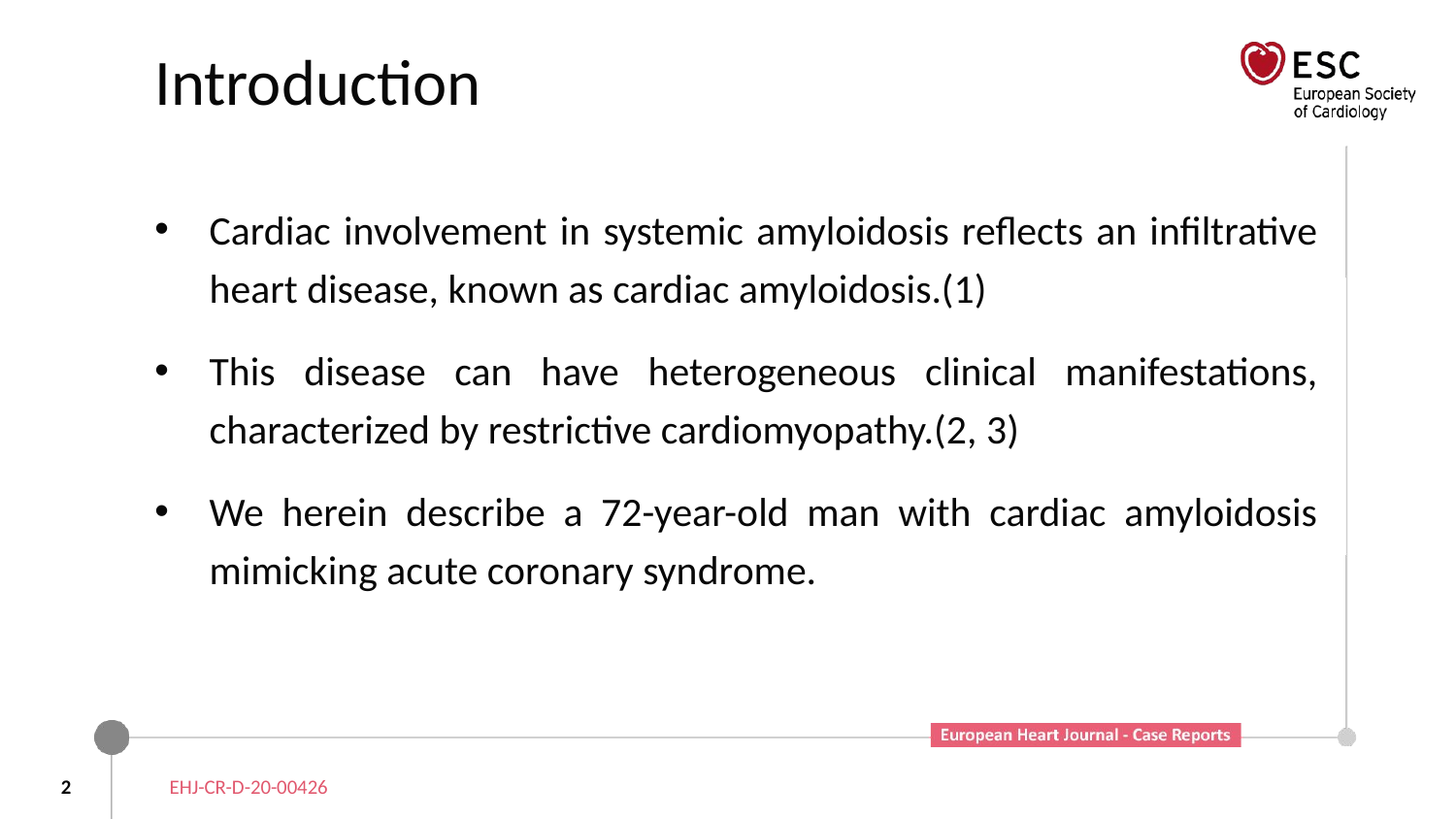

# Introduction
Cardiac involvement in systemic amyloidosis reflects an infiltrative heart disease, known as cardiac amyloidosis.(1)
This disease can have heterogeneous clinical manifestations, characterized by restrictive cardiomyopathy.(2, 3)
We herein describe a 72-year-old man with cardiac amyloidosis mimicking acute coronary syndrome.
2
EHJ-CR-D-20-00426

## Slide 3
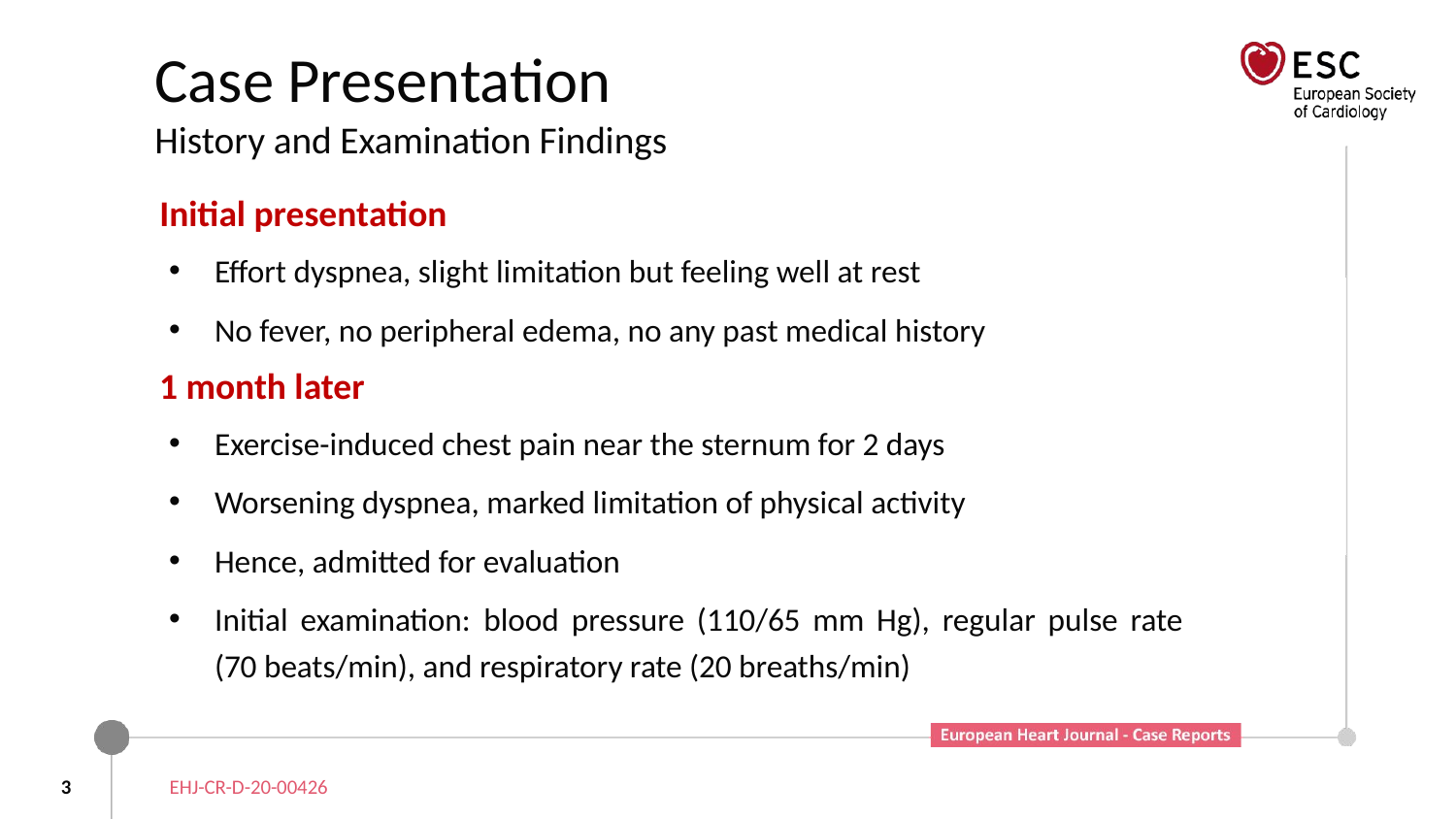

# Case Presentation History and Examination Findings
Initial presentation
Effort dyspnea, slight limitation but feeling well at rest
No fever, no peripheral edema, no any past medical history
1 month later
Exercise-induced chest pain near the sternum for 2 days
Worsening dyspnea, marked limitation of physical activity
Hence, admitted for evaluation
Initial examination: blood pressure (110/65 mm Hg), regular pulse rate (70 beats/min), and respiratory rate (20 breaths/min)
3
EHJ-CR-D-20-00426

## Slide 4
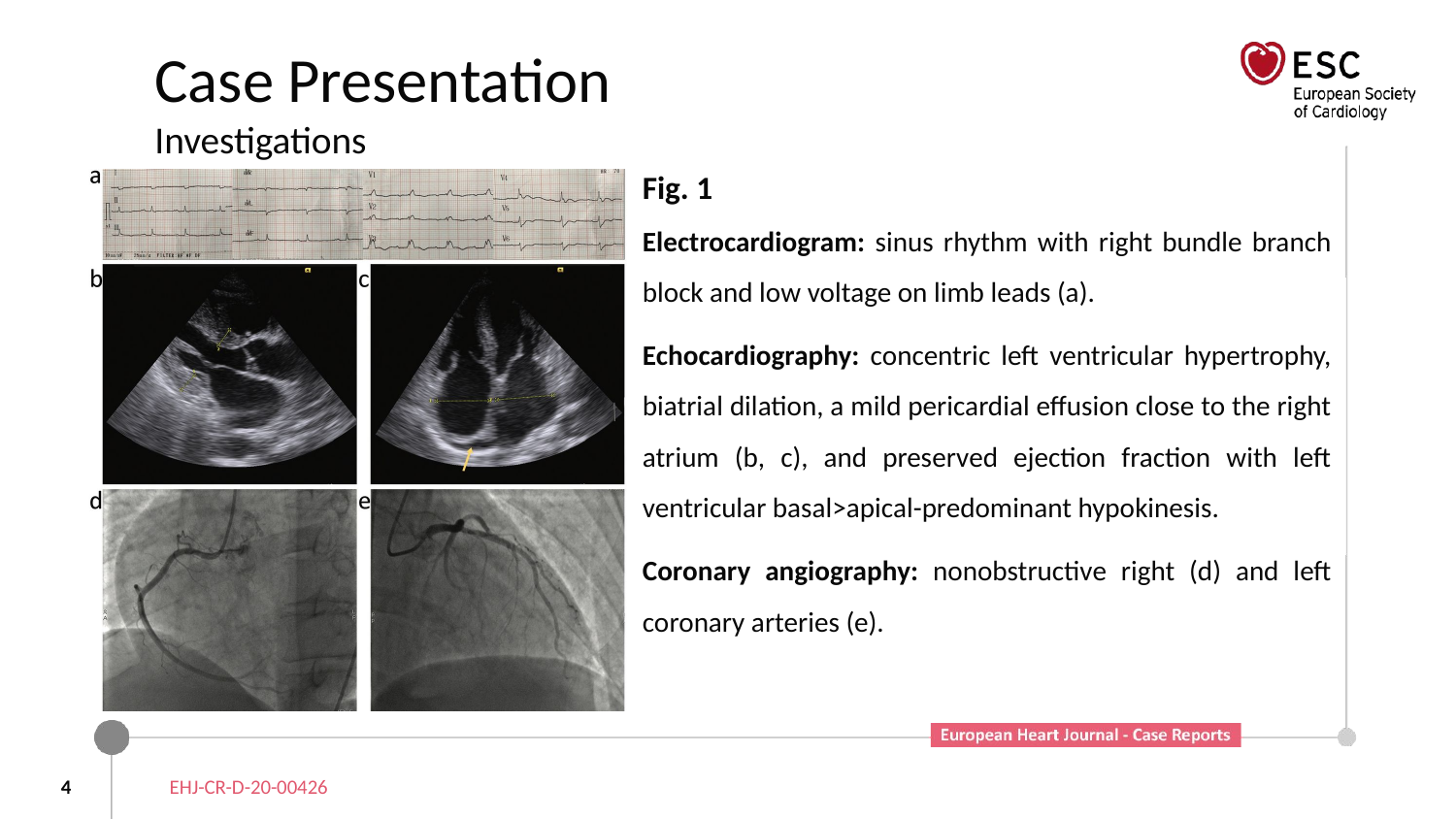

# Case Presentation Investigations
Fig. 1
Electrocardiogram: sinus rhythm with right bundle branch block and low voltage on limb leads (a).
Echocardiography: concentric left ventricular hypertrophy, biatrial dilation, a mild pericardial effusion close to the right atrium (b, c), and preserved ejection fraction with left ventricular basal>apical-predominant hypokinesis.
Coronary angiography: nonobstructive right (d) and left coronary arteries (e).
4
EHJ-CR-D-20-00426

## Slide 5
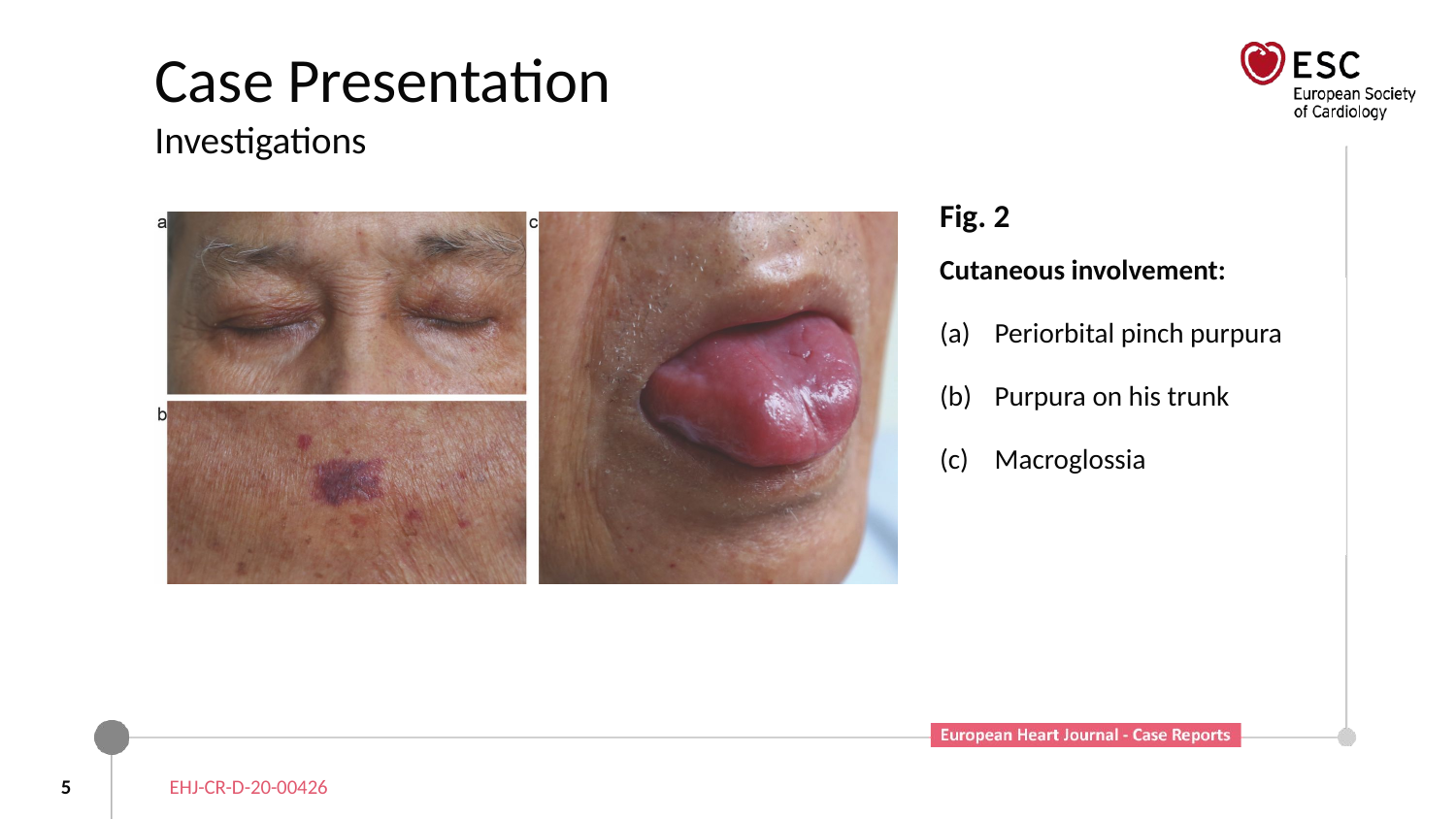

# Case Presentation Investigations
Fig. 2
Cutaneous involvement:
Periorbital pinch purpura
Purpura on his trunk
Macroglossia
5
EHJ-CR-D-20-00426

## Slide 6
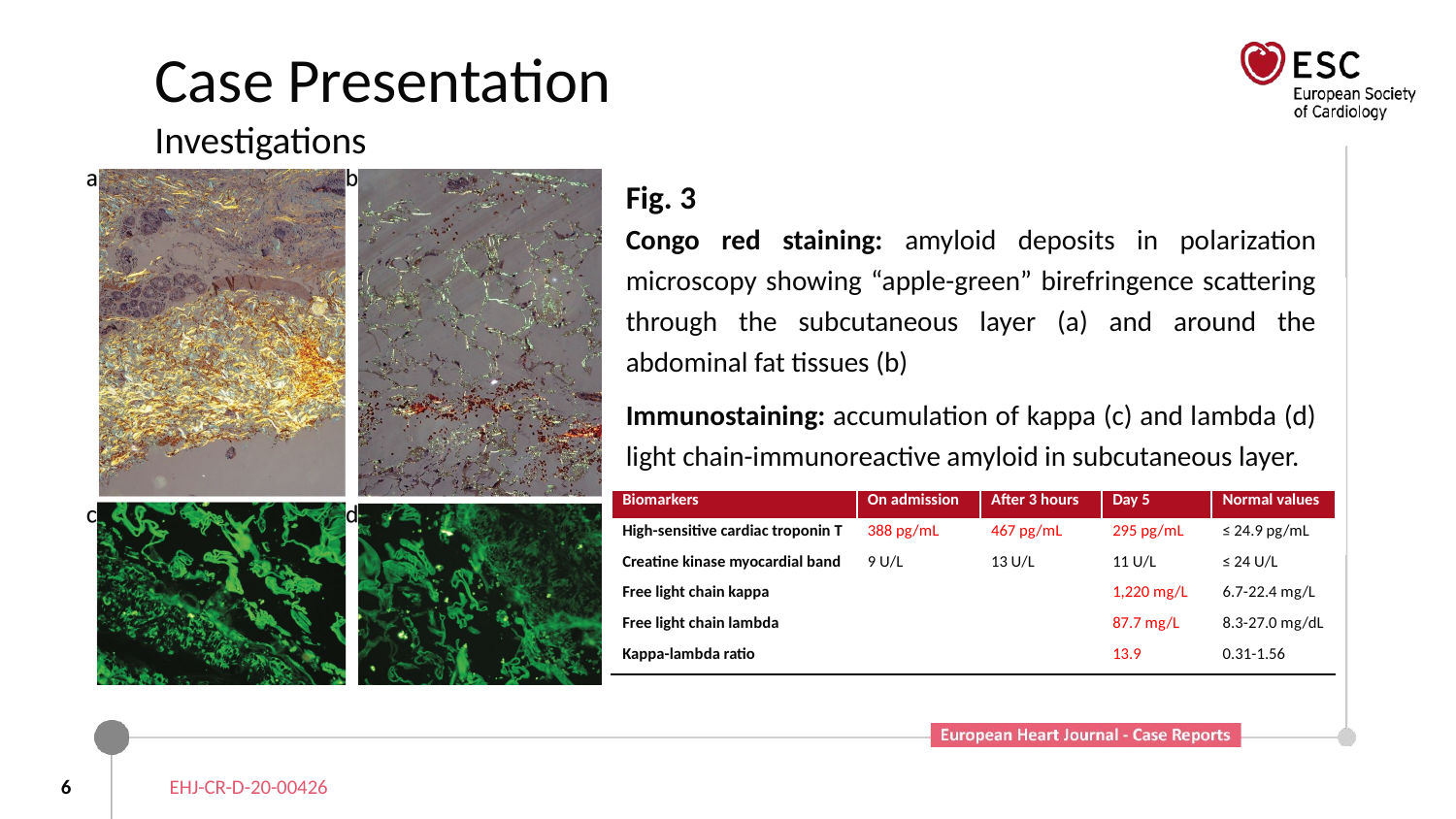

# Case Presentation Investigations
Fig. 3
Congo red staining: amyloid deposits in polarization microscopy showing “apple-green” birefringence scattering through the subcutaneous layer (a) and around the abdominal fat tissues (b)
Immunostaining: accumulation of kappa (c) and lambda (d) light chain-immunoreactive amyloid in subcutaneous layer.
| Biomarkers | On admission | After 3 hours | Day 5 | Normal values |
| --- | --- | --- | --- | --- |
| High-sensitive cardiac troponin T | 388 pg/mL | 467 pg/mL | 295 pg/mL | ≤ 24.9 pg/mL |
| Creatine kinase myocardial band | 9 U/L | 13 U/L | 11 U/L | ≤ 24 U/L |
| Free light chain kappa | | | 1,220 mg/L | 6.7-22.4 mg/L |
| Free light chain lambda | | | 87.7 mg/L | 8.3-27.0 mg/dL |
| Kappa-lambda ratio | | | 13.9 | 0.31-1.56 |
6
EHJ-CR-D-20-00426

## Slide 7
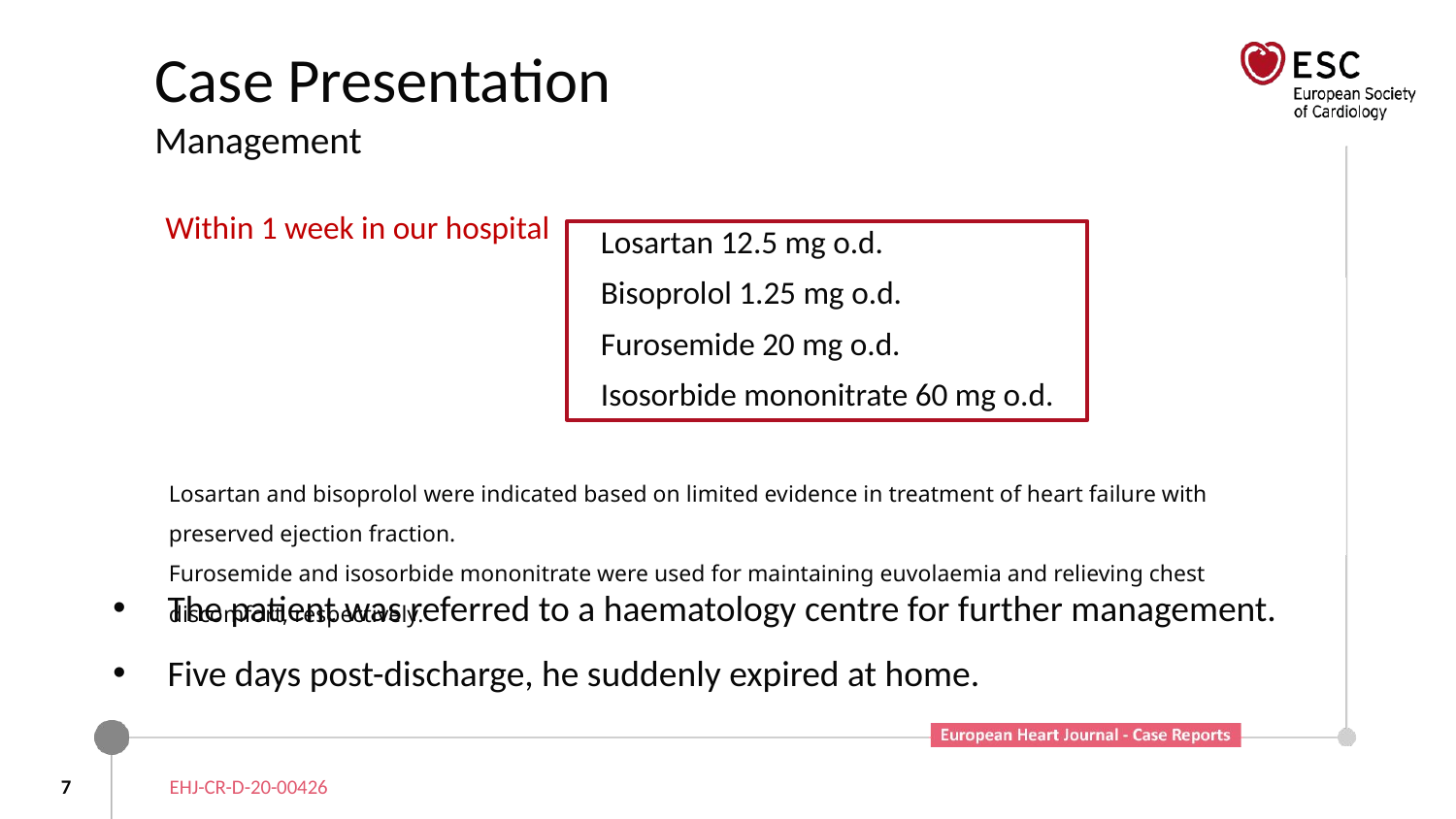

# Case Presentation Management
Within 1 week in our hospital
Losartan 12.5 mg o.d.
Bisoprolol 1.25 mg o.d.
Furosemide 20 mg o.d.
Isosorbide mononitrate 60 mg o.d.
Losartan and bisoprolol were indicated based on limited evidence in treatment of heart failure with preserved ejection fraction.
Furosemide and isosorbide mononitrate were used for maintaining euvolaemia and relieving chest discomfort, respectively.
The patient was referred to a haematology centre for further management.
Five days post-discharge, he suddenly expired at home.
7
EHJ-CR-D-20-00426

## Slide 8
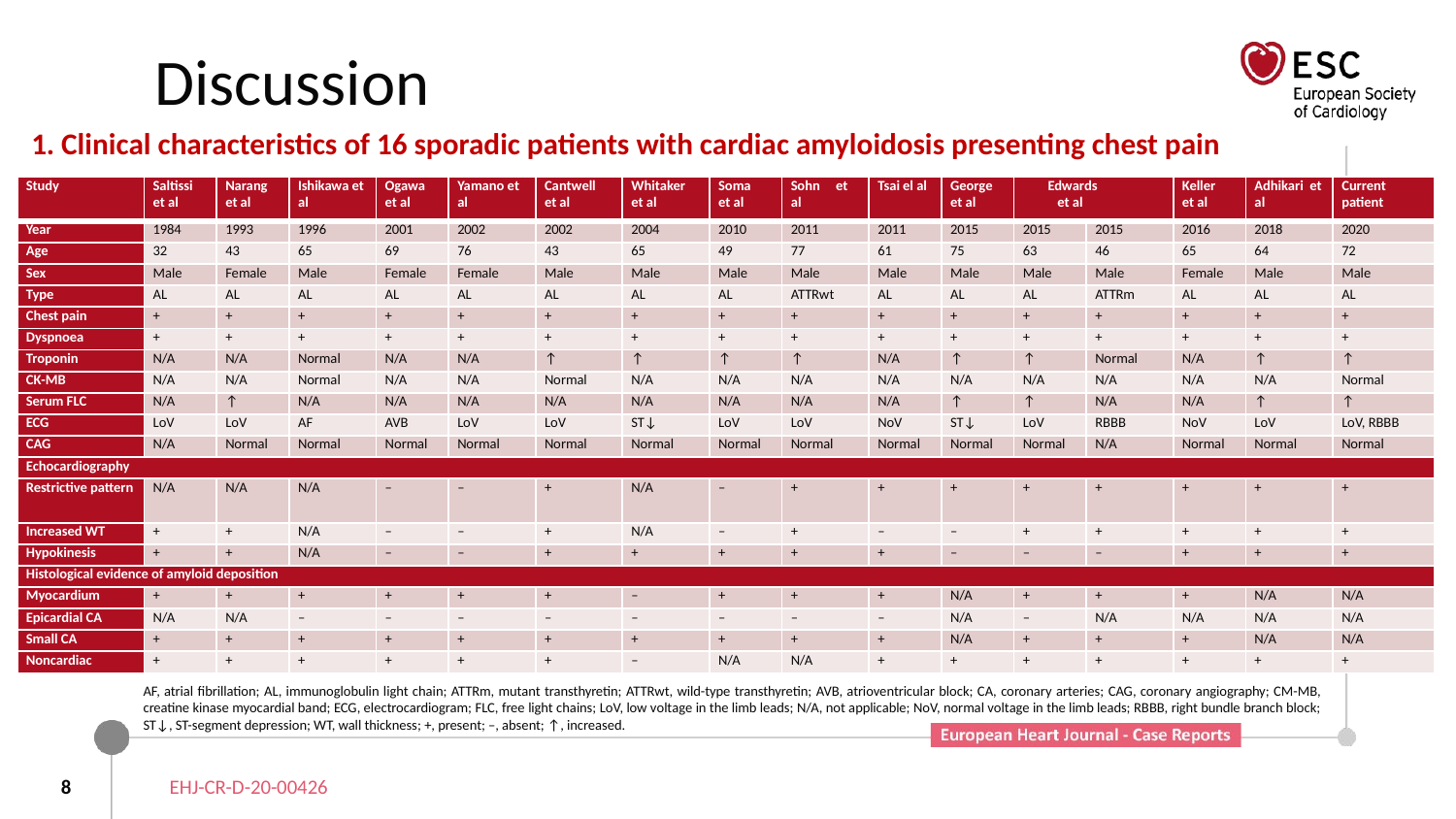

# Discussion
1. Clinical characteristics of 16 sporadic patients with cardiac amyloidosis presenting chest pain
| Study | Saltissi et al | Narang et al | Ishikawa et al | Ogawa et al | Yamano et al | Cantwell et al | Whitaker et al | Soma et al | Sohn et al | Tsai el al | George et al | Edwards et al | | Keller et al | Adhikari et al | Current patient |
| --- | --- | --- | --- | --- | --- | --- | --- | --- | --- | --- | --- | --- | --- | --- | --- | --- |
| Year | 1984 | 1993 | 1996 | 2001 | 2002 | 2002 | 2004 | 2010 | 2011 | 2011 | 2015 | 2015 | 2015 | 2016 | 2018 | 2020 |
| Age | 32 | 43 | 65 | 69 | 76 | 43 | 65 | 49 | 77 | 61 | 75 | 63 | 46 | 65 | 64 | 72 |
| Sex | Male | Female | Male | Female | Female | Male | Male | Male | Male | Male | Male | Male | Male | Female | Male | Male |
| Type | AL | AL | AL | AL | AL | AL | AL | AL | ATTRwt | AL | AL | AL | ATTRm | AL | AL | AL |
| Chest pain | + | + | + | + | + | + | + | + | + | + | + | + | + | + | + | + |
| Dyspnoea | + | + | + | + | + | + | + | + | + | + | + | + | + | + | + | + |
| Troponin | N/A | N/A | Normal | N/A | N/A | ↑ | ↑ | ↑ | ↑ | N/A | ↑ | ↑ | Normal | N/A | ↑ | ↑ |
| CK-MB | N/A | N/A | Normal | N/A | N/A | Normal | N/A | N/A | N/A | N/A | N/A | N/A | N/A | N/A | N/A | Normal |
| Serum FLC | N/A | ↑ | N/A | N/A | N/A | N/A | N/A | N/A | N/A | N/A | ↑ | ↑ | N/A | N/A | ↑ | ↑ |
| ECG | LoV | LoV | AF | AVB | LoV | LoV | ST↓ | LoV | LoV | NoV | ST↓ | LoV | RBBB | NoV | LoV | LoV, RBBB |
| CAG | N/A | Normal | Normal | Normal | Normal | Normal | Normal | Normal | Normal | Normal | Normal | Normal | N/A | Normal | Normal | Normal |
| Echocardiography | | | | | | | | | | | | | | | | |
| Restrictive pattern | N/A | N/A | N/A | – | – | + | N/A | – | + | + | + | + | + | + | + | + |
| Increased WT | + | + | N/A | – | – | + | N/A | – | + | – | – | + | + | + | + | + |
| Hypokinesis | + | + | N/A | – | – | + | + | + | + | + | – | – | – | + | + | + |
| Histological evidence of amyloid deposition | | | | | | | | | | | | | | | | |
| Myocardium | + | + | + | + | + | + | – | + | + | + | N/A | + | + | + | N/A | N/A |
| Epicardial CA | N/A | N/A | – | – | – | – | – | – | – | – | N/A | – | N/A | N/A | N/A | N/A |
| Small CA | + | + | + | + | + | + | + | + | + | + | N/A | + | + | + | N/A | N/A |
| Noncardiac | + | + | + | + | + | + | – | N/A | N/A | + | + | + | + | + | + | + |
AF, atrial fibrillation; AL, immunoglobulin light chain; ATTRm, mutant transthyretin; ATTRwt, wild-type transthyretin; AVB, atrioventricular block; CA, coronary arteries; CAG, coronary angiography; CM-MB, creatine kinase myocardial band; ECG, electrocardiogram; FLC, free light chains; LoV, low voltage in the limb leads; N/A, not applicable; NoV, normal voltage in the limb leads; RBBB, right bundle branch block; ST↓, ST-segment depression; WT, wall thickness; +, present; –, absent; ↑, increased.
8
EHJ-CR-D-20-00426

## Slide 9
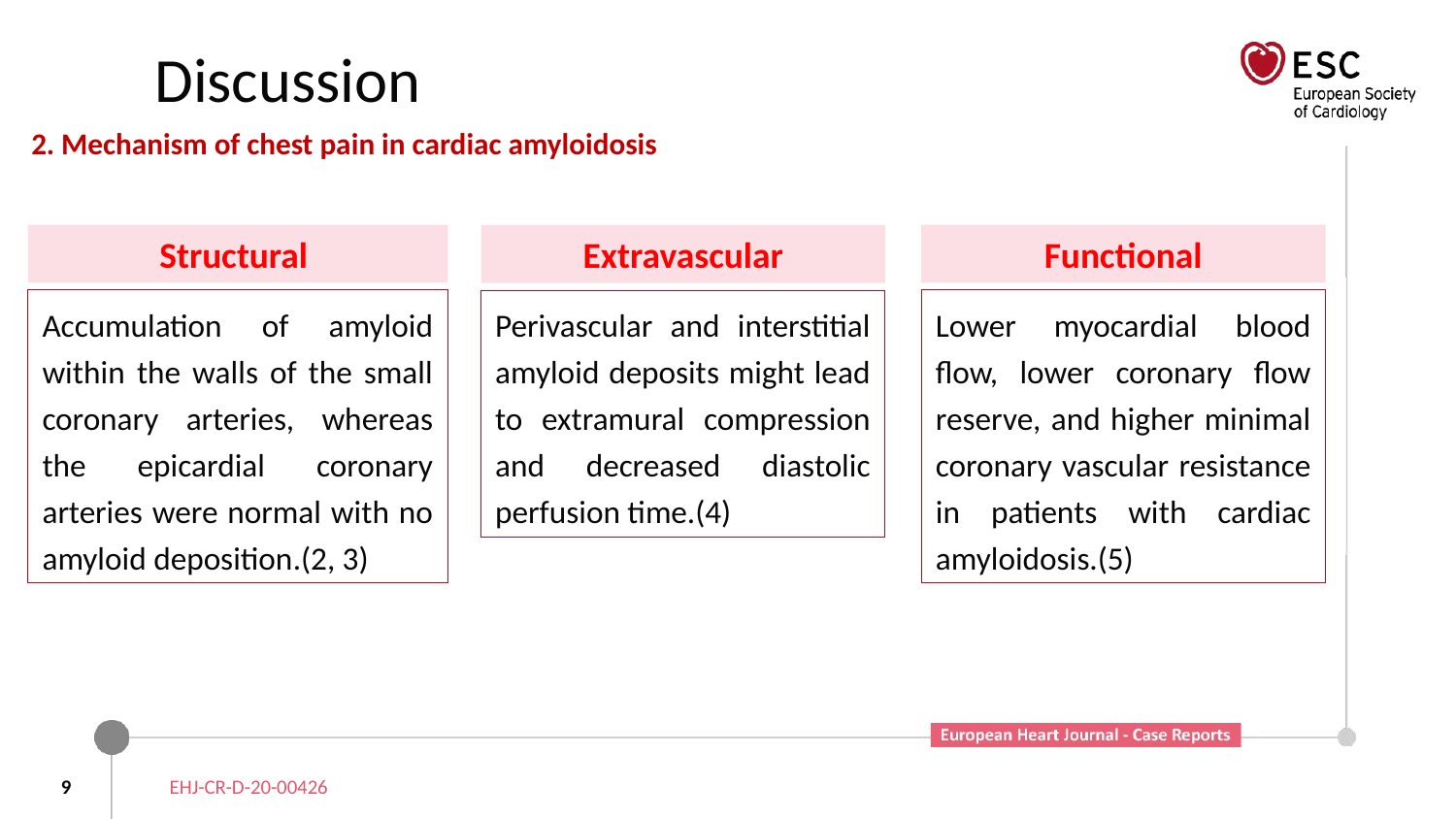

# Discussion
2. Mechanism of chest pain in cardiac amyloidosis
Structural
Functional
Extravascular
Accumulation of amyloid within the walls of the small coronary arteries, whereas the epicardial coronary arteries were normal with no amyloid deposition.(2, 3)
Lower myocardial blood flow, lower coronary flow reserve, and higher minimal coronary vascular resistance in patients with cardiac amyloidosis.(5)
Perivascular and interstitial amyloid deposits might lead to extramural compression and decreased diastolic perfusion time.(4)
9
EHJ-CR-D-20-00426

## Slide 10
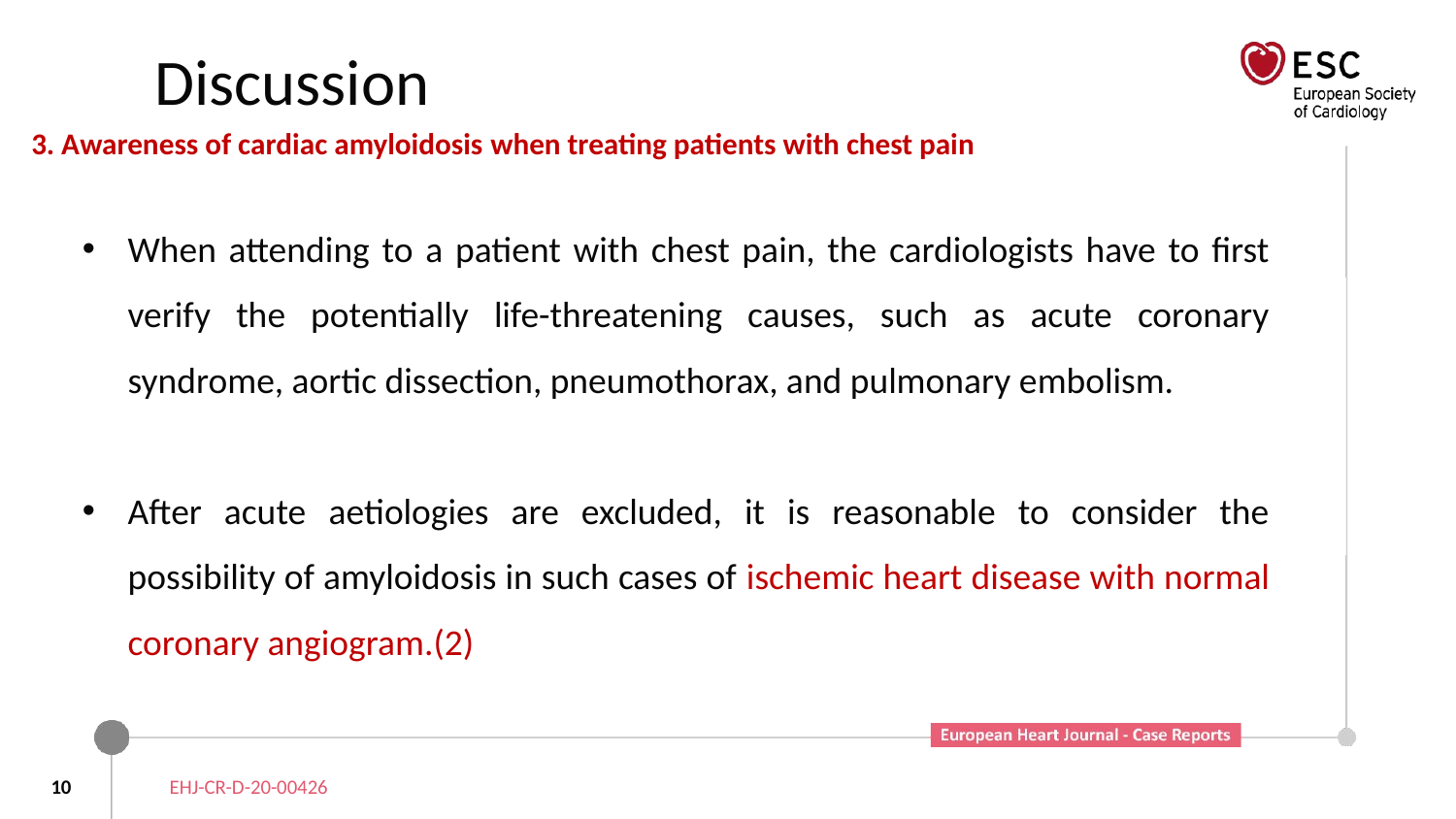

# Discussion
3. Awareness of cardiac amyloidosis when treating patients with chest pain
When attending to a patient with chest pain, the cardiologists have to first verify the potentially life-threatening causes, such as acute coronary syndrome, aortic dissection, pneumothorax, and pulmonary embolism.
After acute aetiologies are excluded, it is reasonable to consider the possibility of amyloidosis in such cases of ischemic heart disease with normal coronary angiogram.(2)
10
EHJ-CR-D-20-00426

## Slide 11
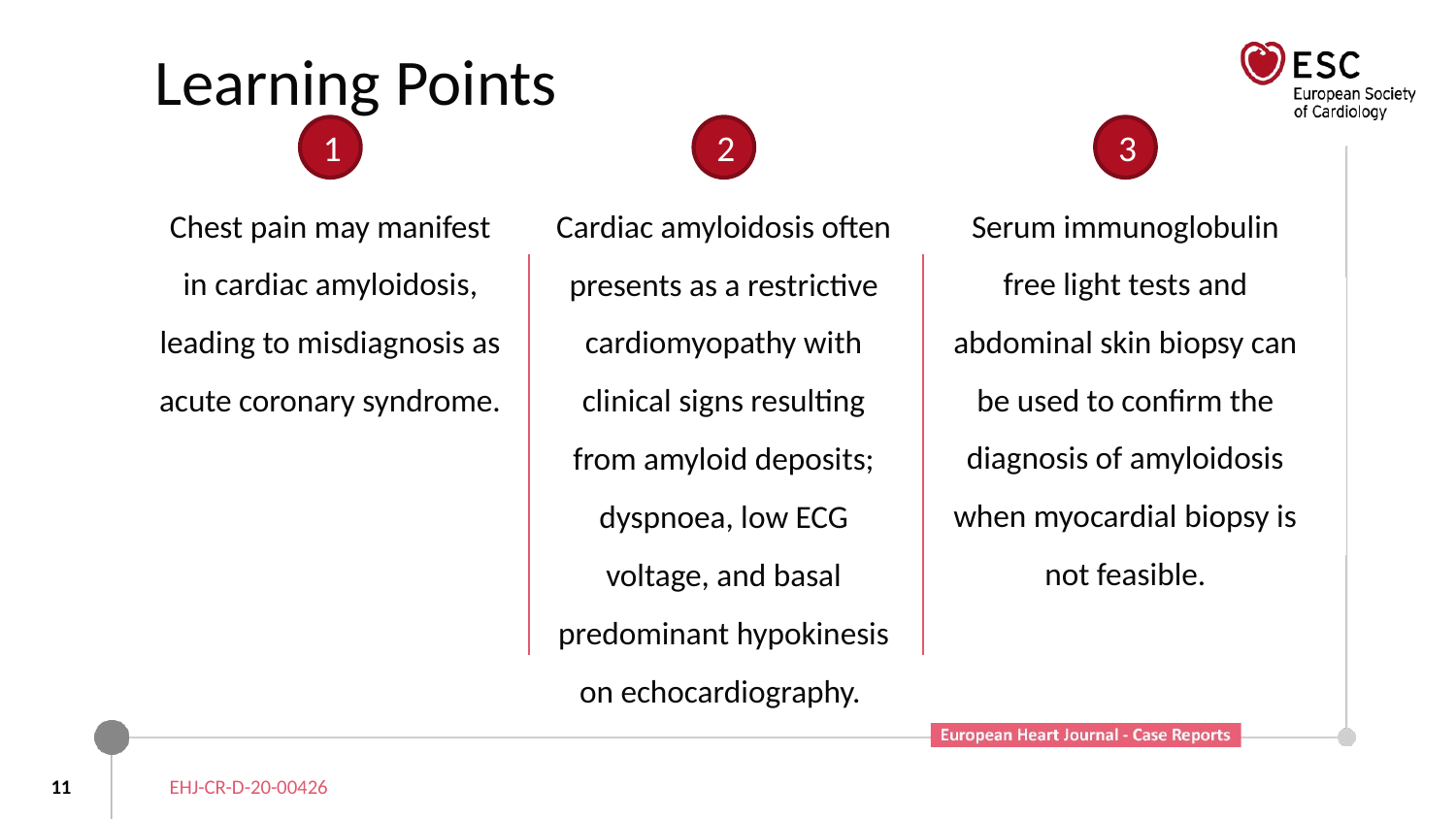

# Learning Points
1
2
3
Chest pain may manifest in cardiac amyloidosis, leading to misdiagnosis as acute coronary syndrome.
Cardiac amyloidosis often presents as a restrictive cardiomyopathy with clinical signs resulting from amyloid deposits; dyspnoea, low ECG voltage, and basal predominant hypokinesis on echocardiography.
Serum immunoglobulin free light tests and abdominal skin biopsy can be used to confirm the diagnosis of amyloidosis when myocardial biopsy is not feasible.
11
EHJ-CR-D-20-00426

## Slide 12
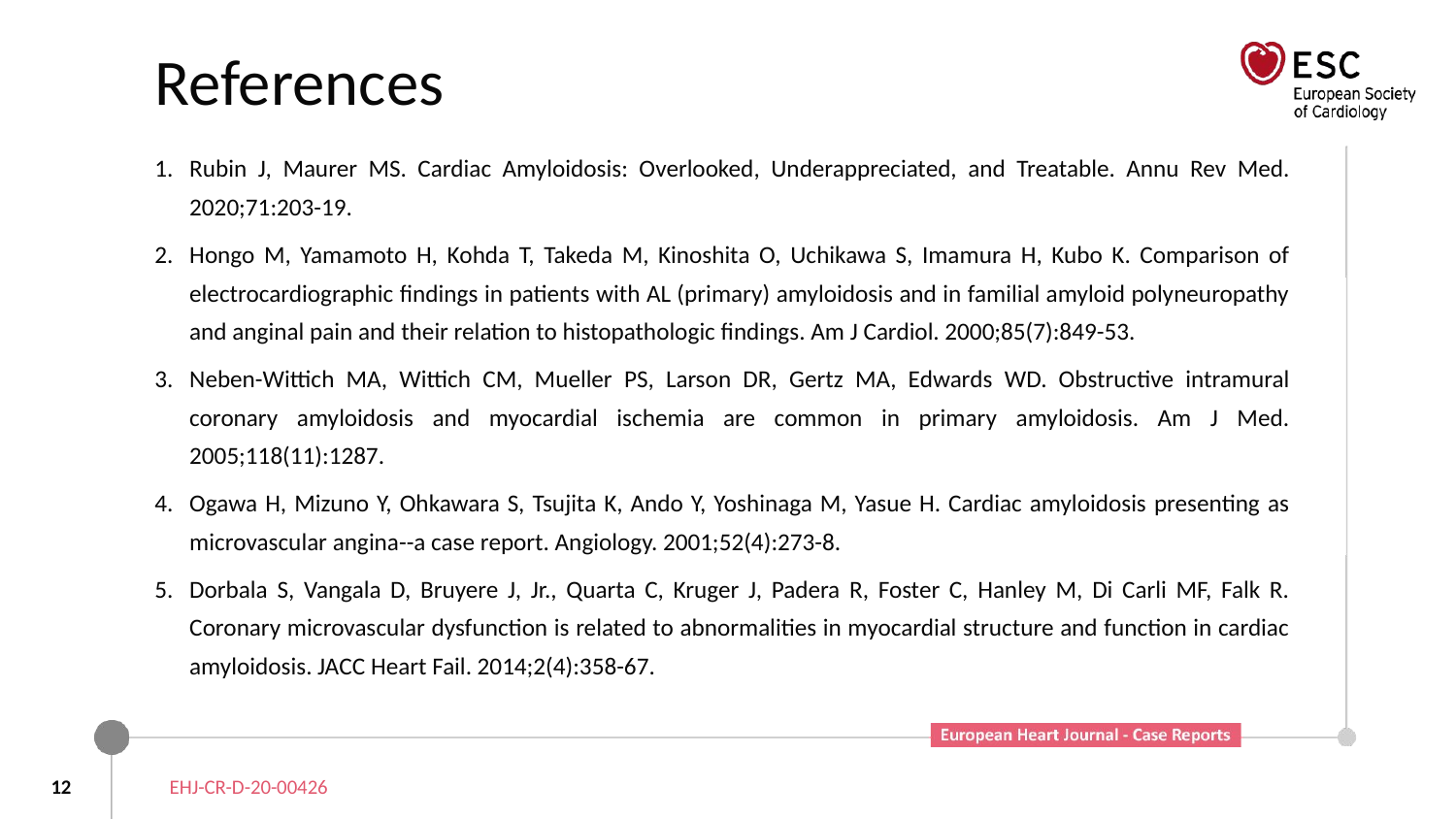

# References
Rubin J, Maurer MS. Cardiac Amyloidosis: Overlooked, Underappreciated, and Treatable. Annu Rev Med. 2020;71:203-19.
Hongo M, Yamamoto H, Kohda T, Takeda M, Kinoshita O, Uchikawa S, Imamura H, Kubo K. Comparison of electrocardiographic findings in patients with AL (primary) amyloidosis and in familial amyloid polyneuropathy and anginal pain and their relation to histopathologic findings. Am J Cardiol. 2000;85(7):849-53.
Neben-Wittich MA, Wittich CM, Mueller PS, Larson DR, Gertz MA, Edwards WD. Obstructive intramural coronary amyloidosis and myocardial ischemia are common in primary amyloidosis. Am J Med. 2005;118(11):1287.
Ogawa H, Mizuno Y, Ohkawara S, Tsujita K, Ando Y, Yoshinaga M, Yasue H. Cardiac amyloidosis presenting as microvascular angina--a case report. Angiology. 2001;52(4):273-8.
Dorbala S, Vangala D, Bruyere J, Jr., Quarta C, Kruger J, Padera R, Foster C, Hanley M, Di Carli MF, Falk R. Coronary microvascular dysfunction is related to abnormalities in myocardial structure and function in cardiac amyloidosis. JACC Heart Fail. 2014;2(4):358-67.
12
EHJ-CR-D-20-00426
