# Supplementary material for: Cardiac amyloidosis mimicking acute coronary syndrome: a case report and literature review
Source: Eur Heart J Case Rep. 2020 Oct 29;4(6):1–7. doi: 10.1093/ehjcr/ytaa325 (PMC7793193; doi:10.1093/ehjcr/ytaa325)
Supplement: ytaa325_Supplementary_Data [file ytaa325_supplementary_data.zip › ytaa325-suppl_data/10_EHJ-CR-D-20-00151R3_Supplementary.doc]

**Supplementary Information**

**Cardiac amyloidosis mimicking acute coronary syndrome:**

**A case report and literature review**

**Systemic literature review**

We identified 6 published case-series **(Supplementary Table S1)** and 15 eligible sporadic cases. Including our case, clinical information of 16 sporadic patients with cardiac amyloidosis presenting chest pain is shown in **Table 1**. The patients had a mean (± standard deviation) age of 60.3 ± 13.6 years with male predominance (12 patients, 75%). All patients experienced dyspnoea. Out of 9 patients (56.3%) that had low voltage in ECG, 7 manifested increased wall thickness. Ten patients (62.5%) revealed basal myocardial predominant hypokinesis in echocardiography. Coronary angiography was done in 14 patients and all of them showed normal epicardial coronary arteries. The serum FLC test was performed in 5 patients and all of them had an elevated serum FLC level. All patients were confirmed systemic amyloidosis by histological evidence of amyloid deposition in the myocardium and/or abdominal skin biopsy. Out of 16 patients, fourteen patients (87.5%) were AL amyloidosis, one (6.25%) was mutant transthyretin (ATTRm) amyloidosis, and one (6.25%) was wild-type transthyretin (ATTRwt) amyloidosis. The data suggest that angina symptoms are more common with AL and may have distinctive mechanisms in the different types of amyloidosis.

**Supplementary Table S1:**  References of case-series.

| 1 | Buja LM, Khoi NB, Roberts WC. Clinically significant cardiac amyloidosis. Clinicopathologic findings in 15 patients. Am J Cardiol. 1970;26(4):394-405. |
| --- | --- |
| 2 | Wright JR, Calkins E. Clinical-pathologic differentiation of common amyloid syndromes. Medicine (Baltimore). 1981;60(6):429-48. |
| 3 | Al Suwaidi J, Velianou JL, Gertz MA, Cannon RO, 3rd, Higano ST, Holmes DR, Jr., Lerman A. Systemic amyloidosis presenting with angina pectoris. Ann Intern Med. 1999;131(11):838-41. |
| 4 | Mueller PS, Edwards WD, Gertz MA. Symptomatic ischemic heart disease resulting from obstructive intramural coronary amyloidosis. Am J Med. 2000;109(3):181-8. |
| 5 | Hongo M, Yamamoto H, Kohda T, Takeda M, Kinoshita O, Uchikawa S, Imamura H, Kubo K. Comparison of electrocardiographic findings in patients with AL (primary) amyloidosis and in familial amyloid polyneuropathy and anginal pain and their relation to histopathologic findings. Am J Cardiol. 2000;85(7):849-53. |
| 6 | Neben-Wittich MA, Wittich CM, Mueller PS, Larson DR, Gertz MA, Edwards WD. Obstructive intramural coronary amyloidosis and myocardial ischemia are common in primary amyloidosis. Am J Med. 2005;118(11):1287. |

**Supplementary Table S2:** References of sporadic cases in Table 1

| 1 | Saltissi S, Kertes PJ, Julian DG. Primary cardiac amyloidosis in a young man presenting with angina pectoris. Br Heart J. 1984;52(2):233-6. |
| --- | --- |
| 2 | Narang R, Chopra P, Wasir HS. Cardiac amyloidosis presenting as ischemic heart disease. A case report and review of literature. Cardiology. 1993;82(4):294-300. |
| 3 | Ishikawa Y, Ishii T, Masuda S, Asuwa N, Kiguchi H, Hirai S, Murayama A. Myocardial ischemia due to vascular systemic amyloidosis: a quantitative analysis of autopsy findings on stenosis of the intramural coronary arteries. Pathol Int. 1996;46(3):189-94. |
| 4 | Ogawa H, Mizuno Y, Ohkawara S, Tsujita K, Ando Y, Yoshinaga M, Yasue H. Cardiac amyloidosis presenting as microvascular angina--a case report. Angiology. 2001;52(4):273-8. |
| 5 | Yamano S, Motomiya K, Akai Y, Shiiki H, Toyama T, Hashimoto T, Fujimoto S, Nakamura S, Fukuoka Y. Primary systemic amyloidosis presenting as angina pectoris due to intramyocardial coronary artery involvement: a case report. Heart Vessels. 2002;16(4):157-60. |
| 6 | Cantwell RV, Aviles RJ, Bjornsson J, Wright RS, Freeman WK, Oh JK, Hoyer JD, Markovic S, Jaffe AS. Cardiac amyloidosis presenting with elevations of cardiac troponin I and angina pectoris. Clin Cardiol. 2002;25(1):33-7. |
| 7 | Whitaker DC, Tungekar MF, Dussek JE. Angina with a normal coronary angiogram caused by amyloidosis. Heart. 2004;90(9):e54. |
| 8 | Soma K, Takizawa M, Uozumi H, Kobayakawa N, Takemura T, Shiraishi J, Aoyagi T. A Case of ST-elevated myocardial infarction resulting from obstructive intramural coronary amyloidosis. Int Heart J. 2010;51(2):134-6. |
| 9 | Sohn HR, Song BG, Jeong SY, Hong SM, Jung HG, Jung HJ, Cho WH, Choi SK. A Case of Cardiac Amyloidosis Initially Misdiagnosed as Syndrome X. Cardiol Res. 2011;2(4):181-4. |
| 10 | Tsai SB, Seldin DC, Wu H, O'Hara C, Ruberg FL, Sanchorawala V. Myocardial infarction with "clean coronaries" caused by amyloid light-chain AL amyloidosis: a case report and literature review. Amyloid. 2011;18(3):160-4. |
| 11 | George A, McClements B. Cardiac amyloidosis presenting as recurrent acute coronary syndrome with unobstructed coronary arteries: Case report. Indian Heart J. 2015;67(6):570-3. |
| 12 | Edwards A, Paulsen M, Lasky B, Ramanathan V. Cardiac amyloidosis: a case review series. Journal of Integrative Cardiology. 2015;1(2):40-5. |
| 13 | Keller FA, Jamshidi P, Boeck BD, Glatz K, Nowack M. Cardiac Amyloidosis Presenting With Myocardial Ischemia: Case Report and Literature Review. Journal of Medical Cases. 2016;7:531-5. |
| 14 | Adhikari BK, Wang Y, Li B, Liu Q, Zhang W. A Case Report of Cardiac Amyloidosis Presenting with Chest Discomfort. Scientific Research Publishing. 2018;8:162-8. |
